# Supplementary material for: Hybrid Models and Biological Model Reduction with PyDSTool
Source: PLoS Comput Biol. 2012 Aug 9;8(8):e1002628. doi: 10.1371/journal.pcbi.1002628 (PMC3415397; doi:10.1371/journal.pcbi.1002628)
Supplement: Text S4 — Complete source code for the PyDSTool package (version 0.88.120504). Includes API documentation and help files linking to web pages. This file is identical to the current public release on Sourceforge.net. (ZIP) [file pcbi.1002628.s004.zip › PyDSTool/html/PyDSTool.Toolbox.optimizers.step.goldstein_price_step-pysrc.html]

xml version="1.0" encoding="ascii"?


PyDSTool.Toolbox.optimizers.step.goldstein\_price\_step


| Home | Trees | Indices | Help | PyDSTool |
| --- | --- | --- | --- | --- |

Package PyDSTool ::
Package Toolbox ::
Package optimizers ::
Package step ::
Module goldstein\_price\_step

[hide private]

[frames] | no frames]

# Source Code for Module PyDSTool.Toolbox.optimizers.step.goldstein\_price\_step

```
 1   
 2  # Matthieu Brucher 
 3  # Last Change : 2007-08-31 14:01 
 4   
 5  """ 
 6  Computes Goldstein-Price step for a specific function at a specific point 
 7  """ 
 8   
 9  import math 
10  from numpy import dot 
11  from numpy.linalg import solve as n_solve 
12  from numpy.linalg import norm, cholesky 
13   


14 -class GoldsteinPriceStep(object):


15    """ 
16    The Goldstein-Price step 
17    """ 


18 -  def __init__(self, mu = 0.1):


19      """ 
20      """ 
21      self.mu = mu 
22      self.nu = math.cos(math.pi/2.0 - mu)

23   


24 -  def __call__(self, function, point, state):


25      """ 
26      Computes Goldstein-Price step  
27      """ 
28      g = function.gradient(point) 
29      state['gradient'] = g 
30      G = function.hessian(point) 
31      state['hessian'] = G 
32   
33      isPositiveDefinite = True 
34      d0 = None 
35   
36      try: 
37          L = cholesky(G) 
38          d0 = n_solve(L.T, n_solve(L, -g)) 
39      except: 
40          isPositiveDefinite = False 
41   
42      if isPositiveDefinite: 
43          cosTheta = dot(d0, -g) / (norm(d0) * norm(g)) 
44          if cosTheta >= self.nu: 
45              step = d0 
46          else: 
47              step = -g 
48      else: 
49          step = -g 
50   
51      state['direction'] = step 
52      return step

53
```

  


| Home | Trees | Indices | Help | PyDSTool |
| --- | --- | --- | --- | --- |

|  |  |
| --- | --- |
| Generated by Epydoc 3.0.1 on Fri May 4 15:24:26 2012 | http://epydoc.sourceforge.net |
